# Women’s overall satisfaction with health facility delivery services in Ghana: a mixed-methods study

**DOI:** 10.1186/s41182-019-0172-7

**Published:** 2019-07-05

**Authors:** Kwame K. Adjei, Kimiyo Kikuchi, Seth Owusu-Agyei, Yeetey Enuameh, Akira Shibanuma, Evelyn Korkor Ansah, Junko Yasuoka, Kwaku Poku-Asante, Sumiyo Okawa, Margaret Gyapong, Charlotte Tawiah, Abraham Rexford Oduro, Evelyn Sakeah, Doris Sarpong, Keiko Nanishi, Gloria Quansah Asare, Abraham Hodgson, Masamine Jimba, Ghana EMBRACE Implementation Research Project Team, Yoshiharu Yoneyama, Ebenezer Appiah-Denkyira, Masamine Jimba, Abraham Hodgson, Gloria Quansah Asare, Evelyn Korkor Ansah, Junko Yasuoka, Keiko Nanishi, Akira Shibanuma, Kimiyo Kikuchi, Sumiyo Okawa, Margaret Gyapong, Sheila Addei, Vida Kukula, Doris Sarpong, Clement Narh, Seth Owusu-Agyei, Kwaku Poku-Asante, Ernest Nettey, Charlotte Tawiah, Yeetey Enuameh, Kwame K. Adjei, Solomon Narh-Bana, Emmanuel Mahama, Francis Dzabeng, Abraham Rexford Oduro, John Williams, Cornelius Debpuur, Francis Yeji, Evelyn Sakeah, Peter Wontuo, Akiko Hagiwara, Sakiko Shiratori, Yusuke Kamiya, Enoch Oti Agyekum

**Affiliations:** 1 Kintampo Health Research Centre, Kintampo, Brong-Ahafo Ghana; 2 Institute of Decision Science for a Sustainable Society, Kyushu University, Fukuoka, Japan; 3 University of Health and Allied Science, Ho, Ghana; 4 Kwame Nkrumah University of Science and Technology, Kumasi, Ghana; 5 Department of Community and Global Health, Graduate School of Medicine, The University of Tokyo, 7-3-1, Hongo, Bunkyo-ku, Tokyo, 113-0033 Japan; 6 Research and Education Center for Prevention of Global Infectious Diseases of Animals, Tokyo University of Agriculture and Technology, Tokyo, Japan; 7 Navrongo Health Research Centre, Navrongo, Ghana; 8 Dodowa Health Research Centre, Dodowa, Greater Accra Ghana; 9 Office of International Academic Affairs, Graduate School of Medicine and Faculty of Medicine, The University of Tokyo, Tokyo, Japan; 10 Ghana Health Service, Accra, Ghana; 11 Research and Development Division, Ghana Health Service, Accra, Ghana

**Keywords:** Maternal health, Child health, Pregnancy, Client satisfaction, Ghana, Mixed methods

## Abstract

**Background:**

Skilled birth delivery has increased up to nearly 74% in Ghana, but its quality has been questioned over the years. As understanding women’s satisfaction could be important to improving service quality, this study aimed to determine what factors were associated with women’s overall satisfaction with delivery services quantitatively and qualitatively in rural Ghanaian health facilities.

**Results:**

This cross-sectional, mixed methods study used an explanatory sequential design across three Ghana Health Service research areas in 2013. Participants were women who had delivered in the preceding 2 years. Two-stage random sampling was used to recruit women for the quantitative survey. Relationships between women’s socio-demographic characteristics and their overall satisfaction with health facility delivery services were examined using univariate and multiple logistic regression analyses. For qualitative analyses, women who completed the quantitative survey were purposively selected to participate in focus group discussions. Data from the focus group discussions were analyzed based on predefined and emerging themes. Overall, 1130 women were included in the quantitative analyses and 136 women participated in 15 focus group discussions. Women’s mean age was 29 years. Nearly all women (94%) were satisfied with the overall services received during delivery. Women with middle level/junior high school education [adjusted odds ratio (AOR) = 0.50, 95% confidence interval (CI) = (0.26–0.98)] were less likely to be satisfied with overall delivery services compared to women with no education. Qualitatively, women were not satisfied with the unconventional demands, negative attitude, and unavailability of healthcare workers, as well as the long wait time.

**Conclusions:**

Although most women were satisfied with the overall service they received during delivery, they were not satisfied with specific aspects of the health services; therefore, higher quality service delivery is necessary to improve women’s satisfaction. Additional sensitivity training and a reduction in work hours may also improve the experience of clients.

## Background

The provision of delivery services by skilled health personnel is vital to improving maternal health [1]; however, between 2012 and 2017, about 50% of deliveries in sub-Saharan Africa (SSA) were performed by skilled personnel, compared to the global picture of 80% deliveries during the same period [2]. In Ghana, about 74% of women had skilled deliveries, compared to 97% who received antenatal care (ANC) from skilled personnel in 2014 [3]. Available evidence suggests that various factors contribute to this gap in service utilization, one of which is client satisfaction with quality of service [4–6]. Client satisfaction is one aspect of adequate service provision, and its influence is currently being assessed in relation to reproductive and maternal healthcare in developing countries [4, 7, 8]. Client satisfaction can be indicative of the quality of the service provided and it is influenced by factors such as cost and staff members’ attitude, among others [9–12]. The assessment of client satisfaction is vital in ensuring that clients receive quality care and that all services are client-oriented [9–12].

The Ghana Ensure Mothers and Babies Regular Access to Care (EMBRACE) implementation research project was launched in 2012 as part of efforts to improve the continuum of care in maternal, newborn, and child health in Ghana [13]. In the framework of the project, current practices on aspects of maternal, newborn, and child health indicators were examined [14]. In those studies, women’s satisfaction with ANC services was suggested as a driving factor in their decision to deliver at a health facility with skilled health personnel [15]. While several studies have concentrated on general patient satisfaction and quality of health services in Ghana, the country does not have much information specifically on clients’ satisfaction with delivery services, and most of the studies conducted in this area used quantitative designs [16]. In their systematic review of qualitative factors and barriers to facility-based delivery, however, Bohren and colleagues identified the need for quantitative findings to be explored in more depth through qualitative approaches [17].

As part of the EMBRACE evaluation to support the work done on satisfaction with delivery services in Ghana and sub-Saharan Africa, we used mixed methods to examine women’s overall satisfaction with delivery services at health facilities in rural Ghana and identified the perceived factors that affected satisfaction with current services.

## Results

### Women’s characteristics

Table 1 highlights the characteristics of 1130 participants who delivered in a health facility; 94% of participants were satisfied with overall care received during facility delivery. Their mean age was 29.0 (SD 9.0) years.

**Table 1:** Women’s socio-demographic characteristics

Characteristics	*N* = 1130	%
Background characteristics of all participants		
Age (years)		
< 20	99	8.8
20–29	570	50.4
30–39	377	33.4
≥ 40	76	6.7
Unknown	8	0.7
Education level		
None	386	34.2
Primary	253	22.4
Middle/junior high school	355	31.4
Secondary	103	9.1
Tertiary and above	33	2.9
Marital status		
Married	718	63.5
Cohabiting	272	24.1
Divorced	37	3.3
Never married	103	9.1
Religion		
Christian	598	53.0
Islamic	132	11.7
Traditional	285	25.2
Other	58	5.1
Missing	57	5.0
Socio-economic characteristics		
Wealth quintile		
Least	227	20.1
Less	233	20.5
Wealthy	221	19.7
Wealthier	223	19.8
Wealthiest	226	20.0
Valid health insurance card possession		
Yes	588	52.0
No	542	48.0
Antenatal history		
Timing of pregnancy		
Got pregnant at the right time	676	59.8
Wanted to get pregnant later	353	31.3
Did not want to get pregnant	10	8.9
Parity		
1	319	28.2
2–3	475	42.1
4–5	224	19.8
> 5	112	9.9
Background characteristics of FGD participants	*N* = 136	%
Age (years)		
< 20	16	11.8
20–29	58	42.6
30–39	50	36.8
≥ 40	12	8.8
Education level		
None	49	36.0
Primary	35	25.7
Middle/junior high school	41	30.2
Secondary	9	6.6
Tertiary and above	2	1.5
Marital status		
Married	90	66.1
Cohabiting	31	22.8
Divorced	7	5.2
Widowed	1	0.7
Never Married	7	5.2
Religion		
Christian	71	55.0
Islamic	17	13.2
Traditional	33	25.6
Other	8	6.2

### Determinants of women’s satisfaction with overall health facility delivery services

In the univariate analysis, two independent variables were associated with overall satisfaction with health facility-based delivery services: education level and timing of pregnancy. In the multiple logistic regression analysis, education level was significantly associated with overall satisfaction with delivery services. Women with middle level/junior high school education [adjusted odds ratio (AOR) = 0.50, 95% confidence interval (CI) = (0.26–0.98)] were less likely to be satisfied with overall delivery services compared to women with no education (Table 2).

**Table 2:** Determinants of women’s overall satisfaction with health facility delivery services

Characteristics	Crude OR	*P* value	95% CI	Adjusted OR	*P* value	95% CI
Education						
None	Ref			Ref		
Primary	0.91	0.76	0.49–1.69	0.87	0.65	0.47–1.61
Middle/junior high	0.48	0.02*	0.25–0.91	0.50	0.04*	0.26–0.98
Secondary	0.52	0.29	0.16–1.75	0.55	0.35	0.16–1.91
Tertiary and above	0.34	0.04*	0.12–0.94	0.38	0.06	0.14–1.04
Timing of pregnancy						
Satisfied with timing	Ref			Ref		
Wanted to get pregnant later	2.02	0.04*	1.02–3.99	1.92	0.06	0.98–3.76
Did not want to get pregnant	0.82	0.65	0.35–1.92	0.70	0.42	0.30–1.65
Complications during most recent delivery						
Yes	Ref			Ref		
No	1.57	0.10	0.92–2.67	1.54	0.11	0.91–2.60

### Qualitative study findings

The background characteristics of the focus group discussions (FGD) respondents are described in Table 1. Two main themes emerged from the FGDs: satisfaction and dissatisfaction with delivery services. These themes were made up of sub-themes with greater details provided below (Table 3).

**Table 3:** Themes and sub-themes identified in focus group discussions

Themes	Sub-themes
Dissatisfaction	Long wait time
	Negative attitude of health workers
	Unconventional demands from health workers
	Low availability of health workers
	Inconsistent treatment by health workers
Satisfaction	Good treatment from health workers
	Encouragement from health workers

#### Dissatisfaction with health facility delivery services

##### Long wait time

Some women stated that they were not satisfied with their delivery at the health facilities because of the long wait time. Two women explained their dissatisfaction as follows:

“ … When we got there, there was nobody around. After settling down, we went to call them [health worker]; but, they did not come. We waited for a very long time” (19-year-old woman, Dodowa).

“ … When I was going to give birth, they sent me to a health center and I waited for a long time. It was not until the nurses who give the folders realized that I was suffering; so, they looked for a car and sent me back to Navrongo to deliver” (28-year-old woman, Navrongo).

##### Negative attitude of health workers

Most women were not satisfied with the services received from health workers during delivery, especially the attitude of the health workers during the labor. This was explained by two women as follows:

“When I was in pain, instead of the nurses taking care of me, they talked to me harshly. They are also women; so, they should understand what you are going through. Instead, they do things that they are not supposed to do such as talking to you harshly” (25-year-old woman, Dodowa).

“ … As for nurses when you go to them, they are supposed to be patient. However, I experienced problems with my pregnancy and I expected them to be patient with me; but, they were not. It was also my first pregnancy, and, instead of them to be patient and tell me what to do, they were shouting. Meanwhile, helping women to deliver is part of their work” (22-year-old woman, Navrongo).

##### Unconventional demands from health workers

One woman explained her dissatisfaction due to unconventional demands from health workers:

“When I was in labor and got to the hospital, they (health workers) asked me to go and jump and do some exercise. The little strength I had, they told me to do all sort of things and when I became weak and unable to give birth, they yelled at me and had me transferred. These are problems that affects us, and a lot of people prefer to give birth at home” (30-year-old woman, Dodowa).

##### Unavailability of health workers at facility

Some women stated that health workers were not available at the facility during their time of delivery:

“ … When we got to the facility, the woman who was to help me deliver was not there” (27-year-old woman, Navrongo).

##### Inconsistent treatment by health workers

Some women stated that they were not satisfied with the inconsistent treatment received by different staff members during delivery. Asked to explain further, one participant summarized it as follows:

“ … Some are always patient with us; but, others, they are not at all. What she is supposed to do to make everybody happy, she won’t at all. She shouts at you like a child. They are not all the same...When I came to the facility to deliver, some were good to me, and some were not” (24-year-old woman, Dodowa)*.*

### Satisfaction with health facility delivery services

#### Satisfaction with health workers

While most women were not satisfied with health workers, some women were completely satisfied with the health workers. They described them as people who gave respect, showed empathy, and provided good treatment during delivery. One woman had this to say regarding this:

“ … They (health workers) really respect and treat you well when you go to give birth too. They know how to pamper you till you deliver and after delivery too they continue. The way they dress up your baby and also treat the mothers are very good. They even educated me on how to take care of myself before I went home” (30-year-old woman, Navrongo).

#### Encouragement from health workers

Aside from being satisfied with the treatment received during delivery, these women spoke highly of the health workers who attended to them. They were particularly grateful for the encouragement they received from health workers. One woman explained further:

“If your time is due for delivery, the nurses here motivate you and tell you to get courage to deliver safely. They even go ahead to give time of delivery. Unlike other places, during delivery, they don’t shout at you” (32-year-old woman, Kintampo).

## Discussion

Nearly all women who delivered were satisfied with the overall care they received during delivery at health facilities, according to the quantitative findings of the present study. The qualitative findings provide in-depth information on the various aspects of delivery services they were satisfied or dissatisfied with.

The quantitative results showed a relationship between education and satisfaction with delivery services, which both supports and contrasts prior literature. Montasser and colleagues from their study conducted in Egypt found no relationship between education level and satisfaction with delivery services [7]. Colombara and colleagues, on the other hand, from their study conducted in Central America noted that women with at least a primary education or higher were less likely to be satisfied with delivery services than those with no education [18]. This could be because these women expect to be given preferential treatment, which may also be the case in the present study.

The univariate logistic regression model used in our analysis showed that women were also satisfied or dissatisfied based on the timing of their pregnancy. Although this was not statistically significant in the multiple logistic regression model, it is consistent with other available publications on women’s satisfaction with delivery services. Women who have unintended or unwanted pregnancies are less likely to be satisfied with delivery services [4, 19, 20]. This is attributed to the fact that these women are generally not happy and are worried about bringing up their newborn. These women may also experience other issues such as depression, lack of family support, or domestic abuse due to the fact that the pregnancy was unwanted [21].

The qualitative findings showed that that women’s dissatisfaction with facility delivery services was influenced primarily by the behavior of health workers. This finding supports related work on satisfaction with quality of health services conducted in Ghana and other sub-Saharan countries. The poor attitudes of health workers in Ghana remain a major challenge as highlighted by Asamani and colleagues [22]. Recent reports by SEND-Ghana as well as Duku and colleagues on healthcare quality show that the bad attitude of health workers is often aimed particularly toward clients under the National Health Insurance Scheme (NHIS) [23, 24]. Ghanaian women who deliver at a health facility are not supposed to pay for the services provided as they are registered under NHIS [25]. The scheme was introduced in 2003 with the primary aim of ensuring that all registered residents have access to basic health services [26, 27]. Free maternal healthcare was introduced under the NHIS in 2008 after an initial maternal exemption fee policy in 2005 and encourages women to access maternal health services such as ANC and delivery services at no cost to them [28–30]. There is growing evidence, however, that the NHIS is not always working as effectively as it should, resulting in non-availability of some drugs at health facilities [31, 32]. In addition, even though the NHIS has improved access to health care, health workers have been accused of giving better services to patients who are able to pay for their drugs themselves, as compared to those who rely solely on the NHIS for payment [23].

Regarding workers’ attitudes, some women revealed in the FGDs that, when health workers had shown respect and empathy toward them, it contributed immensely to their satisfaction with delivery services. This is consistent with the findings of earlier studies. Empathy from health workers has been shown to be a major contributor to satisfaction with delivery services among women who deliver in these health facilities [33, 34], and in a study on satisfaction conducted by Bazant and colleagues among Kenyans, empathy from service providers such as health workers had a positive impact on patients’ satisfaction [20]. Hodnett and colleagues conducted a systematic review of randomized controlled trials and found that women who experience complications during pregnancy value empathy from health workers and are therefore more likely to be satisfied with the delivery [35, 36]. They also showed that women who have continuous support during delivery have a shorter labor period and are less likely to be dissatisfied with delivery services [35, 36].

Finally, our study highlighted the important role played by health workers in determining women’s satisfaction with facility delivery services, which has a number of policy implications. Although not conclusive, our findings potentially support the need for a change in health workers’ attitudes toward women during delivery. This is in line with the findings and recommendations by Asamani et al. in their study on poor attitudes of health workers, where they identified the need for sensitivity training for health workers, particularly when they assist patients during delivery [28]. This study also found that the Ghana Health Service (GHS) code of ethics as well as the code of conduct and disciplinary procedures were not being enforced in the health system [22]. These codes outline disciplinary measures for poor attitude of staff toward clients [37, 38]. More stringent measures are needed to enforce these codes, such as occasional monitoring and supervision by heads of health facilities to correct and maintain staff behavior. Official channels should be made available at the health facilities for clients to express such grievances. At the same time, clients should also be empowered to report such incidents of problematic behavior to prevent them from reoccurring.

There might be a need to increase the number of health professionals, especially midwives, at health facilities as the ratio of midwives to patients is quite low (nine midwives per 10,000 patients) [39]. The World Health Organization (WHO) recommends a ratio of at least 40 nursing and midwifery personnel per 10,000 [40]. Even though Ghana in 2016 had a nursing and midwifery personnel ratio of 33.7 per 10,000, nurses contributed a greater percent (over 90%) compared with midwives [40]. Ghana’s nursing and midwifery ratio was also lower than that of other neighboring SSA countries such as Cote D’Ivoire (81.1 per 10,000) and Liberia (67.1 per 10,000) within that period [40]. Task-strengthening of lower-level staff, such as community health officers/nurses (CHOs/CHNs) at CHPS, enabling them to aid with deliveries may help in resolving this challenge [41]. Task-sharing has been used previously in Ghana among CHOs in family planning to improve uptake of implants and may reduce staff members’ workloads while improving working conditions, thus improving staff morale and subsequently fostering greater delivery satisfaction among women [42].

### Limitations and strengths

This study had some limitations. First, overall satisfaction relied on women’s reports, which could have introduced social desirability bias, as compared to other questions such as those on education, which were verified using the NHIS cards among others. Second, the primary focus of the quantitative survey was women’s overall satisfaction, and this was binary. This meant that we could not gain a more granular understanding of the concept of quality service. However, the detailed, qualitative aspects of our study contribute to strengthening it. The mixed methods approach allowed for the use of qualitative interviews to explore the quantitative findings, thus providing a more complete understanding of satisfaction with delivery services in the study area. This was also a population-based survey in three different locations in Ghana, which comprised a representative sample of women from the lower, middle, and upper parts of the country who delivered at health facilities. Further, the exploratory aspect (i.e., FGDs) provided a very detailed perspective on why some women from the three study areas were satisfied or dissatisfied with delivery services at the health facilities.

## Conclusion

Although nearly all women were satisfied with the overall service they received at health facilities during delivery, many were not satisfied with the long wait time or the attitudes of the health workers. Consequently, we recommend that these aspects of service delivery be tackled to improve women’s satisfaction with delivery service and, subsequently, quality of care for maternal and child health.

## Methods

### Study design

This cross-sectional, mixed methods study was conducted in 2013 using an explanatory sequential design, which allowed for further investigations into the main quantitative finding using qualitative techniques [43].

### Study area

The EMBRACE study was a collaborative effort between the Ghana Health Service (GHS), the University of Tokyo, and the Japan International Cooperation Agency Institute [13, 44]. The GHS has three health research centers (HRC): Kintampo, Dodowa, and Navrongo. They are located within the three main geographical zones of Ghana, where well-established health and demographic surveillance systems (HDSSs) collect population-based longitudinal data [15, 45]. The Dodowa HRC is located within the Shai-Osu Doku and Ningo-Prampram districts of the Greater Accra region, comprising about 20 health facilities [14, 15]. The Kintampo HRC is in the middle part of Ghana within the Kintampo North Municipality and Kintampo South district in the Brong Ahafo region [46]. In this area, over 40 health facilities provide health services to a predominantly rural population [14, 15, 46]. The Navrongo HRC is in the Kassena-Nankana East and West districts of the Upper East region [47]. The center is renowned for the introduction of the community-based health planning and services concept and has over 50 health facilities [14, 42, 47].

### Sampling procedures and data collection

Women (*N* = 1500) who had delivered in the preceding 2 years were interviewed using a structured questionnaire for the quantitative aspect of this study. Two-stage random sampling was used to recruit 500 women for each HDSS site [44]. Depending on the HDSS site, the primary sampling unit (PSU) was either the sub-district or the community-based health planning and services (CHPS) zone. The CHPS zones/sub-districts are regarded as the lowest health administration units in Ghana. The study areas contained 22 PSUs; women were randomly selected from each CHPS zone/sub-district using probability proportional to size. Women who delivered live or still births within the past 2 years and were resident in the selected areas from January 2011 to April 2013 were included. These women were then traced to their residence with the aid of the HDSS. The HDSS database provided the district, sub-district, community, and resident number (compound number) of participants. All women who provided written consent to be in the study were then interviewed by trained field workers. Field workers at each of the sites completed 2 days training concerning the questionnaire and other technical aspects of the study.

For the qualitative component, 15 (five per site) focus group discussions (FGDs) were conducted among 136 women who took part in the quantitative survey. For each HDSS site, five communities, which had been included in the quantitative survey, were purposively selected for the interviews. Between eight and 12 women who took part in the quantitative survey were then purposively selected from each of the five communities by the study team. These women were then contacted by the data collectors and a date was scheduled for the FGD. Women who were contacted and were willing to take part in the FGD were interviewed. The interviews were conducted by trained researchers from the three HDSS sites using an interview guide that was developed and pre-tested prior to data collection. These researchers also completed 2 days of intensive training in their respective sites. For each FGD conducted, one researcher served as the moderator and the other as a note taker. The duration of each FGD was 45 to 60 min.

### Data collection tools and variables

A structured questionnaire developed by the Ghana EMBRACE team was used for quantitative data collection. Key maternal neonatal and child health practices from pregnancy to delivery and postnatal care were the main focus of the EMBRACE study and questions on these indicators were incorporated. During questionnaire development, per prior literature, the team incorporated a question about satisfaction with delivery services. Since this was a mixed methods approach with a qualitative dimension, the team focused on women’s overall satisfaction with health facility-based delivery services. This was obtained using a binary question (with a yes/no answer) as used by the Ghana Demographic Health Survey for its population-based surveys [16]. The main question was framed as follows: “Taking into consideration the cleanliness of the health facility you visited during delivery, the waiting time, and the treatment received from staff amongst others, were you satisfied with the services received during your most recent pregnancy at the health facility?”. “That is to say, were you satisfied with your delivery services?” The questionnaire was initially tested among selected participants outside the study area. The questionnaire also included questions on the respondents’ background characteristics such as women’s age, education level, marital status, religion, timing of pregnancy, number of pregnancies, complications during delivery, and place of delivery. Socio-economic status included possession of a valid health insurance card and wealth quintile. The main dependent variable was women’s overall satisfaction with health facility-based delivery services. Wealth quintile was measured using assets and was generated based on the methods used by demographic and health surveys [15].

An FGD guide complemented the survey questionnaire. The interviews were recorded using a digital audio recorder. In addition to other questions, the main question asked in the FGD was, “What accounted for your overall satisfaction or otherwise with the health facility delivery services received during your most recent pregnancy at the health facility?”

### Data analyses

Women’s background characteristics were described and reported as proportions. A univariate logistic regression analysis was conducted to determine the relationship between women’s overall satisfaction with facility delivery services and each independent variable. Further analyses were completed using multiple logistic regression analyses. Out of 1500 data entries, we included 1130 in the analysis, with some excluded because of missing key background information (*n* = 3), duplicate maternal information in the case of twins (*n* = 6), or miscarriage (*n* = 1) [42, 44]. We also excluded women who did not deliver in a health facility (*n* = 360) [15].

The following variables identified by the EMBRACE team from literature were used for the univariate logistic regression model: education, timing of pregnancy, age, marital status, location, possession of a valid insurance card, number of children, satisfaction with ANC, place of delivery, who assisted with the delivery, complications during delivery, and maternal check-up after delivery [4, 7, 16, 20, 48, 49]. For the multiple logistic regression model, variables from the univariate model that were found to have significant association with women’s overall satisfaction at *p* ≤ 0.20 were included. These were education, timing of pregnancy, and complications during the last delivery. The probable variables with multi-collinearity were thus excluded from the analyses and no collinearity was observed among the variables used in the multiple regression model. Statistical significance was set at *p* < 0.05. The clustering observed as a result of the study design was accounted for using generalized estimated equations (GEE). GEE uses robust estimation of standard errors which caters for clustering. All analyses were conducted using Stata version 12 (Stata Corporation, College Station, TX). The reference group options for the logistic regression were not specified but chosen by default in Stata.

For the qualitative component, the running notes from the FGDs were expanded. The audio recordings were also transcribed and read several times to clean the data. Translation of interviews that were conducted in the local language was done during the transcription. The information collected was listed in Microsoft Word 2012. The transcription data were coded according to themes, which were categorized manually to reflect themes. Although predefined themes were formed from the interview guide prior to data collection, some evolved as data analyses progressed. The qualitative analysis focused primarily on perceived factors affecting satisfaction and dissatisfaction with facility delivery services based on participants’ experiences.

## Abbreviations

ANC: Antenatal clinic
aOR: Adjusted odds ratio
CHN: Community health nurse
CHOs: Community health officers
CHPS: Community-based health planning and services
CI: Confidence interval
cOR: Crude odds ratio
EMBRACE: Ensure Mothers and Babies Regular Access to Care
FGD: Focus group discussions
GEE: Generalized estimated equation
GHS: Ghana Health Service
HDSS: Health and demographic surveillance systems
HRC: Health research centres
IEC: Institutional ethics committee
IRB: Institutional review board
NHIS: National Health Insurance Scheme
PSU: Primary sampling unit
RDD: Research and development division
REC: Research ethics committee
SD: Standard deviation
SSA: Sub-Saharan Africa
WHO: World Health Organization
